# Provincial distribution and factors associated with high completed fertility among married and cohabiting women aged 40–49 years in Sierra Leone: a cross-sectional study

**DOI:** 10.1093/inthealth/ihae058

**Published:** 2025-02-17

**Authors:** Augustus Osborne, Alhaji Mustapha Abu, Hassan S Rogers, Florence Gyembuzie Wongnaah, Bright Opoku Ahinkorah

**Affiliations:** Department of Biological Sciences, School of Basic Sciences, Njala University, PMB, Freetown 0232, Sierra Leone; Department of Biological Sciences, School of Basic Sciences, Njala University, PMB, Freetown 0232, Sierra Leone; Department of Public Health, Ernest Bai KoromaUniversity of Science and Technology, Makeni Campus, Makeni 0232, Sierra Leone; Department of Global Public Health, Karolinska Institutet, Stockholm 171 77, Sweden; REMS Consultancy Services Limited, Sekondi-Takoradi, Western Region 0233, Ghana; Faculty of Health and Medical Sciences, The University of Adelaide, Adelaide SA 5005, Australia

**Keywords:** Demographic and Health Survey, high completed fertility, public health, Sierra Leone, women

## Abstract

**Background:**

High completed fertility among married and cohabiting women has significant implications, such as burden on resources, exacerbating healthcare issues and educational and gender disparities. This study examined the provincial distribution and factors associated with high completed fertility among married and cohabiting women aged 40–49 y in Sierra Leone.

**Methods:**

Data for the study were sourced from the 2019 Sierra Leone Demographic and Health Survey. Our study comprised 2253 married and cohabiting women aged 40–49 y in Sierra Leone. Geographic variations in high fertility were presented using a spatial map. A mixed-effect multilevel binary logistic regression analysis was performed to identify the factors associated with high completed fertility. The findings were presented as adjusted ORs (aOR) with 95% confidence intervals (CIs).

**Results:**

The national prevalence of high completed fertility among married and cohabiting women in Sierra Leone was 61.7% (58.9–64.5). Women whose partners had secondary/higher education (aOR=0.54, 95% CI 0.33 to 0.89) had lower odds of high completed fertility than those with no formal education. Women in the Northern province (aOR=0.39, 95% CI 0.17 to 0.87) had lower odds of high completed fertility than those in the Eastern province. Women who indicated **≥**6 as their ideal number of children had a higher (aOR=8.10, 95% CI 4.58 to 14.35) likelihood of experiencing high completed fertility compared with those whose ideal number of children was 0–3. Those who were using contraceptives at the time of the survey had a higher (aOR=2.09, 95% CI 1.28 to 3.41) likelihood of having high completed fertility compared with those who were not using contraceptives. Women in the poorer (aOR=1.70, 95% CI 1.07 to 2.72) and middle wealth index quintiles (aOR=2.09, 95% CI 1.29 to 3.41) had higher odds of high completed fertility than those in the poorest wealth index quintile.

**Conclusions:**

A significant proportion (>60%) of married and cohabiting women aged 40–49 y in Sierra Leone have high completed fertility. Partner’s education, province, ideal number of children, use of contraceptives and wealth index were the factors associated with high completed fertility among women in Sierra Leone. Policymakers in Sierra Leone should increase access to and education on family planning methods to empower women to make informed choices about their fertility. The government and policymakers should support educational opportunities, particularly for men, because they are usually the heads of households and can influence fertility decisions. In-depth interviews should be conducted with women who use contraception to understand their motivations and experiences.

## Introduction

High completed fertility is defined as ≥5 births per woman over their reproductive lifespan.^1^ It has negative consequences for both mothers and their children. It increases health risks and hampers investment in human capital. Additionally, it hinders economic growth and worsens environmental challenges.^1^ The worldwide fertility decreased from 3.2 births per woman in 1990 to 2.5 births in 2019.^2^ As of 2021, sub-Saharan Africa had the highest fertility level among all regions with 4.6 children per woman,^2^ with either no fertility falls or a slight decrease.^3^

As of 2023, the worldwide population was about 8 billion and is expected to increase to almost 9.8 billion by 2050.^4^ The global total fertility rate or the average number of lifetime births per woman is 2.2. The rate varies across different regions, with Central Africa having a rate of 5.6 and East Asia having a rate of 1.1.^4^ The United Nations has forecasted that the global population in 2100 will reach 11 billion, while the predicted population for middle African countries is expected to exceed 3.97 billion.^5,6^ The significant rise in the projected population of most sub-Saharan African countries, including those in Middle Africa, can be attributed to a high initial fertility rate that has been gradually declining. The onset of the decrease in fertility in this region was delayed by around 20 years compared with other developing countries.^4^ Furthermore, once the fall began, it is believed to have occurred at a rate that was only one-fourth as fast as in Asia and Latin America at the same stage of demographic development.^7^

The degree of fertility significantly influences reproductive patterns and is influenced by several linked factors, including age, marital status, income, educational level and parity.^8–10^ Various factors, including socioeconomic status, education, mother’s age, the number of children previously born, infant mortality and the views of prominent individuals on intended family size, have been shown to significantly influence decisions about fertility.^11–13^

Since 1974, Sierra Leone has undergone a fertility transition, as evidenced by the decrease in the total fertility reported in the 1974 Population Census, which averaged 6.5 children per woman.^14^ The average number of children per woman in 2015 was 5.2, a continuous decrease. Nevertheless, there has been significant variation in fertility rates among different provinces, ranging from 5.6 children per woman in the Northern province to 5.5 and 5.4 children per woman in the Southern and Eastern provinces, respectively. According to the 2015 Census Analytical Report, the Western province has the lowest total fertility of 4.0 children per woman.^14^ This indicates that the country is starting to experience a decrease in fertility rates, which, if sustained, is likely to lead the country into a demographic dividend.

Sierra Leone recognises high completed fertility as a challenge to development and has implemented various programmes and policies to address it. The government aims to increase access to family planning information and methods through clinics and community outreach programmes.^15^ The Free Health Care for Pregnant Women and Children initiative aims to reduce child mortality, potentially lessening the desire for larger families.^16^ Educational programmes empower girls to delay marriage and make informed choices about childbearing.^14^ Implementing these programmes has faced challenges, such as many rural areas lacking adequate healthcare facilities and trained personnel, limiting access to family planning services.^17^ Traditional beliefs and practices that value large families can hinder the adoption of family planning methods. Government agencies may lack the infrastructure and expertise for efficient programme implementation and monitoring. These challenges create roadblocks for effectively reducing high completed fertility in Sierra Leone.^15^

Previous studies have identified demographic, socioeconomic health-related, cultural, geographical and child-related factors influencing fertility rates in Sierra Leone.^18–24^ For example, Samura et al.^18^ studied the influence of education, wealth index, age, and province on fertility among Sierra Leone women aged 15–49 y, whereas our study examined the association between several factors such as working status, educational level, listening to radio, watching television, reading newspapers or magazines, internet use, ethnicity, and religion and high fertility among women aged 40–49, a demographic that is often overlooked in fertility studies. In this study, we examined how these factors operate in a variety of provincial contexts within Sierra Leone, to gain a deeper understanding of their associations with high completed fertility. Age-specific characteristics are necessary to study reproductive dynamics among older women who may have different socioeconomic and life-stage considerations that may influence their fertility decisions. In this way, Sierra Leone could potentially gain deeper insights and make more tailored policy recommendations to address fertility issues.

## Methods

### Data source

Data for this study were sourced from the 2019 Sierra Leone Demographic and Health Survey (SLDHS), a cross-sectional survey that collects information on demographic, health and nutritional aspects of non-elderly adults and children.^25^ The data collection process utilised structured questionnaires, employing a cross-sectional design and a multistage sampling procedure.^25^ Details about the survey design and methodology have been published in the report.^25^ The study conducted interviews with a total of 15 574 women aged 15–49 y. Our study comprised 2253 married and cohabiting women aged 40–49 y in Sierra Leone. This study followed the Strengthening Reporting of Observational Studies in Epidemiology (STROBE) guidelines.^26^

### Variables

Completed fertility was the outcome variable, defined in the Demographic and Health Survey (DHS) as the total number of children ever born by women aged 40–49 y. We recoded the variable into low and high completed fertility by assigning a value of 1 to individuals with ≥5 children (high) and a value of 0 to individuals who had ≤4 children (low).

### Explanatory variables

Following a detailed literature review on predictors of high completed fertility,^27,28^ we included 16 explanatory variables based on their availability in the SLDHS. The variables were grouped into individual and contextual level variables. The individual level variables consisted of current working status, educational level, listening to radio, watching television, reading newspapers or magazines, internet use, ethnicity, religion, partner’s educational level, ideal number of children, decision-making on healthcare and use of contraceptives. The contextual level variables included wealth index, sex of household head, as well as type of place of residence and province. Table 1 shows the coding scheme of the variables.

**Table 1.** Bnd characteristics of women in Sierra Leone (n=2253)

Variable	Weighted sample (n)	Weighted frequency (%)
**Educational attainment**		
No education	1808	80.2
Primary	200	9.0
Secondary/higher	245	10.8
**Religion**		
Christians	472	21.0
Muslims	1781	79.0
**Current working status**		
Not working	250	11.1
Working	2003	88.9
**Read newspapers or magazines**		
No	2175	96.6
Yes	77	3.4
**Listen to radio**		
No	1345	59.7
Yes	908	40.3
**Watch television**		
No	1806	80.1
Yes	447	19.9
**Use internet**		
No	2141	95.0
Yes	112	5.0
**Ethnicity**		
Fullah	83	3.7
Kono	116	5.1
Limba	203	9.0
Mende	734	32.6
Temne	753	33.4
Korankoh	99	4.4
Other	265	11.7
**Partner’s educational attainment**		
No education	1608	71.3
Primary	139	6.2
Secondary/higher	506	22.5
**Ideal number of children**		
0–3	261	11.6
4–5	781	34.7
≥6	1211	53.7
**Decision on healthcare**		
Respondent	292	13.0
Joint (respondent and partner)	796	35.3
Other (partner alone, someone else)	1165	51.7
**Contraceptive use**		
No	1939	86.1
Yes	314	13.9
**Sex of household head**		
Male	1755	77.9
Female	498	22.1
**Wealth index**		
Poorest	523	23.2
Poorer	492	21.8
Middle	513	22.8
Richer	386	17.1
Richest	339	15.1
**Place of residence**		
Urban	752	33.4
Rural	1 501	66.6
**Province**		
Eastern	536	23.8
Northern	523	23.2
Northwestern	381	16.9
Southern	459	20.4
Western	354	15.7

### Data analyses

The statistical analyses were conducted using Stata software version 17.0 (Stata Corporation, College Station, TX, USA). First, a spatial map was used to show the proportion of women with high completed fertility. Next, we determined the distribution of the explanatory variables across the outcome variable and used a Pearson χ^2^ test to show their associations. Finally, using four models, a mixed-effect multilevel binary logistic regression analysis was conducted to identify the predictors of high completed fertility. Model I, which did not include any explanatory variables, revealed the changes in high completed fertility ascribed to the clustering at the principal sampling units. In model II, the individual level variables were included, while in model III, the contextual level variables were included. Model IV included all the explanatory variables. The mixed-effect regression analysis yielded results that included both fixed effects and random effects. The fixed-effect analysis revealed the correlation between the explanatory predictors and high completed fertility. The results were reported as adjusted ORs (aORs) with their corresponding 95% CIs. The random effect results, however, indicate the variations in high completed fertility. All four models used intracluster correlation coefficient (ICC) values to determine the variation in high completed fertility and help in understanding the extent to which fertility outcomes vary between different clusters (e.g. provinces). All the analyses were weighted, and the svyset command in Stata, which contains the sampling weights, ≥1 stage(s) of clustered sampling and stratification, was used to deal with the complex nature of the DHS dataset. This ensured that the results are representative and account for the survey's design features.

### Ethical considerations

Ethical clearance was not sought for this study because of the publicly available nature of the SLDHS dataset. However, before utilising the dataset, we obtained permission to acquire and utilise the SHDS for publication from Monitoring and Evaluation to Assess and Use Results Demographic and Health Surveys (MEASURE DHS). DHS programmes typically involve review by an Institutional Review Board in the host country, ensuring adherence to local ethical standards, and our study conforms to that standard. Additional details regarding the ethical concerns and principles related to the DHS and its use of data may be found at https://dhsprogram.com/Methodology/Protecting-the-Privacy-of-DHS-Survey-Respondents.cfm.

## Results

### Background characteristics of married and cohabiting women in Sierra Leone

Table 1 shows the background characteristics of high completed fertility among married and cohabiting women in Sierra Leone. While most women in this study (80.2%) had no formal education and identified as Muslim (79.0%), a high proportion (88.9%) were employed. The majority, 95.0%, had never used the internet. Temne was the most common tribe (33.4%). Most of their partners (71.3%) also had no formal education, and 53.7% considered ≥6 children ideal. The majority (86.1%) were not using contraceptives. Socioeconomic factors also play a role, with 23.2% in the poorest category and 66.6% residing in rural areas.

### Prevalence of high completed fertility among married and cohabiting women in Sierra Leone

**Figure 1.**
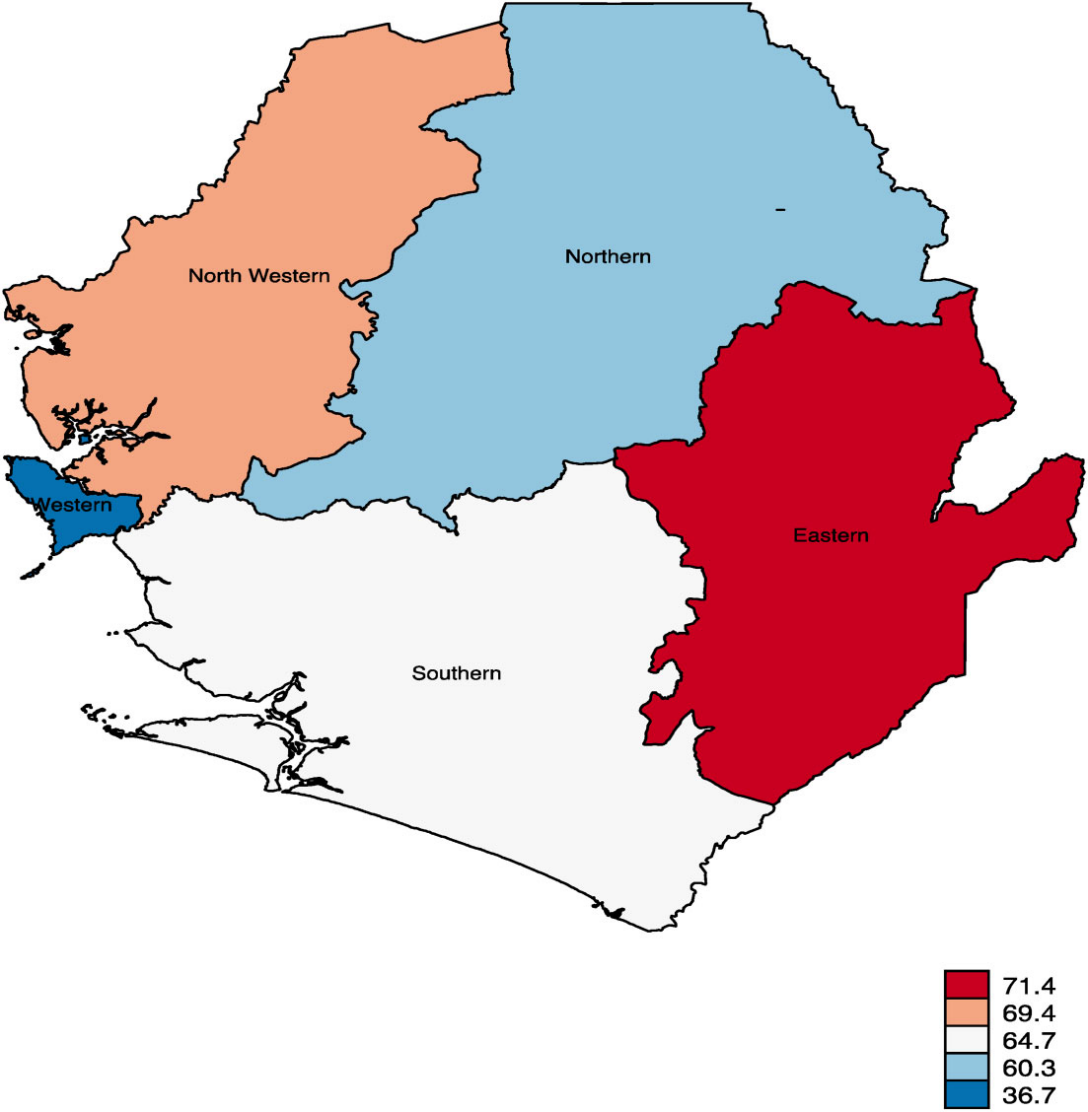
Prevalence of high completed fertility among married and cohabiting women in Sierra Leone.

Figure 1 presents the prevalence of high completed fertility among married and cohabiting women in Sierra Leone. High completed fertility was high among married and cohabiting women living in the Eastern province (71.4%), while married and cohabiting women living in the Western province (36.7%) had the lowest prevalence of high fertility in Sierra Leone. The national prevalence of high completed fertility among married and cohabiting women in Sierra Leone was 61.7 (58.9–64.5) (Table 2).

**Table 2.** Bivariable analysis of high completed fertility among married and cohabiting women in Sierra Leone

Variables	High completed fertility	p-value
**Prevalence**	**61.7% [58.9, 64.5]**	
**Educational attainment**		<0.001
No education	65.3 [62.5, 68.0]	
Primary	65.8 [57.2, 73.5]	
Secondary/higher	31.9 [25.4, 39.2]	
**Religion**		<0.001
Christians	50.4 [43.8, 56.9]	
Muslims	64.7 [61.9, 67.4]	
**Current working status**		0.003
Not working	51.2 [43.4, 58.9]	
Working	63.0 [60.1, 65.9]	
**Read newspapers or magazines**		<0.001
No	63.3 [60.4, 66.1]	
Yes	15.9 [9.1, 26.3]	
**Listen to radio**		0.122
No	63.2 [59.8, 66.6]	
Yes	59.4 [55.4, 63.4]	
**Watch television**		<0.001
No	65.4 [62.6, 68.2]	
Yes	46.7 [40.4, 53.0]	
**Use internet**		<0.001
No	63.8 [60.9, 66.5]	
Yes	22.3 [14.6, 32.7]	
**Ethnicity**		0.004
Fullah	53.0 [42.3, 63.5]	
Kono	68.0 [55.0, 78.7]	
Limba	54.8 [44.6, 64.6]	
Mende	67.7 [63.0, 72.0]	
Temne	61.6 [57.1, 65.9]	
Korankoh	51.3 [41.5, 61.0]	
Other	54.7 [47.5, 61.7]	
**Partner’s educational attainment**		<0.001
No education	66.9 [63.9, 69.8]	
Primary	64.9 [55.7, 73.1]	
Secondary/higher	44.2 [38.3, 50.4]	
**Ideal number of children**		<0.001
0–3	37.7 [30.9, 45.0]	
4–5	44.2 [39.9, 48.6]	
≥6	78.2 [75.2, 80.9]	
**Decision on healthcare**		0.076
Respondent	64.8 [56.5, 72.3]	
Joint (respondent and partner)	57.4 [53.1, 61.7]	
Other (partner alone, someone else)	63.9 [60.0, 67.6]	
**Contraceptive use**		<0.001
No	60.0 [57.0, 63.0]	
Yes	72.1 [66.3, 77.2]	
**Sex of household head**		0.007
Male	63.4 [60.2, 66.5]	
Female	55.7 [50.7, 60.6]	
**Wealth index**		<0.001
Poorest	64.8 [59.9, 69.4]	
Poorer	71.2 [66.4, 75.5]	
Middle	73.3 [68.8, 77.4]	
Richer	58.4 [53.1, 63.5]	
Richest	29.5 [23.0, 36.9]	
**Place of residence**		<0.001
Urban	47.7 [42.3, 53.1]	
Rural	68.8 [65.7, 71.6]	
**Province**		<0.001
Eastern	71.4 [65.9, 76.4]	
Northern	60.3 [54.9, 65.4]	
Northwestern	69.7 [63.7, 75.1]	
Southern	64.7 [59.5, 69.6]	
Western	36.7 [29.5, 44.5]	

P-values were generated from a χ^2^ test.

### Bivariate results of the association between the explanatory variable and total fertility

Table 2 presents the bivariable analysis of the prevalence and distribution of high completed fertility among married and cohabiting women in Sierra Leone. High completed fertility was high among married and cohabiting women who had primary education (65.8%), were Muslims (64.7%), were working (63.0%), who were not using the internet (63.8%), who were Kono by tribe (68.0%), whose partner had no education (66.9%), whose ideal number of children was ≥6 (78.2%), who were using contraceptives (72.1%), were in the middle wealth index (73.3%), lived in rural areas (68.8%) or in the Eastern province (71.4%). Decisions on healthcare and listening to the radio were the only explanatory variables not significantly associated with high completed fertility among married and cohabiting women in Sierra Leone (p<0.05). The remaining variables were statistically significant.

### Factors associated with high completed fertility among married and cohabiting women in Sierra Leone

#### Fixed effect results

Table 3 shows the factors associated with high completed fertility among married and cohabiting women in Sierra Leone. The results of the final model (Model IV) which had all the explanatory variables are reported here. Women whose partners had secondary/higher education (aOR=0.54, 95% CI 0.33 to 0.89) had lower odds of high completed fertility than those with no formal education. Women in the Northern province (aOR=0.39, 95% CI 0.17 to 0.87) had lower odds of high completed fertility than those in the Eastern province. Women who indicated ≥6 as their ideal number of children had a higher (aOR=8.10, 95% CI 4.58 to 14.35) likelihood of high completed fertility compared with those whose ideal number of children was 0–3. Those who were using contraceptives at the time of the survey had a higher (aOR=2.09, 95% CI 1.28 to 3.41) likelihood of high completed fertility compared with those who were not using contraceptives. Women in the poorer (aOR=1.70, 95% CI 1.07 to 2.72) and middle wealth index quintiles (aOR=2.09, 95% CI 1.29 to 3.41) had higher odds of high completed fertility than those in the poorest wealth index quintile.

**Table 3.** Factors associated with high completed fertility among married and cohabiting women in Sierra Leone

	Model I	Model II	Model III	Model IV
Variables	Empty model	aOR [95% CI]	aOR [95% CI]	aOR [95% CI]
**Fixed effect results**				
**Educational attainment**				
No education		1.00		1.00
Primary		1.36 [0.74, 2.48]		1.32 [0.72, 2.42]
Secondary/higher		0.45^*^ [0.22, 0.91]		0.51 [0.25, 1.05]
**Religion**				
Christians		1.00		1.00
Muslims		1.17 [0.71, 1.92]		1.10 [0.67, 1.79]
**Current working status**				
Not working		1.00		1.00
Working		1.35 [0.74, 2.45]		1.28 [0.70, 2.36]
**Read newspaper or magazine**				
No		1.00		1.00
Yes		0.29 [0.06, 1.28]		0.33 [0.07, 1.54]
**Watch television**				
No		1.00		1.00
Yes		1.32 [0.77, 2.26]		1.56 [0.90, 2.71]
**Use internet**				
No		1.00		1.00
Yes		0.99 [0.35, 2.81]		1.13 [0.40, 3.15]
**Ethnicity**				
Fullah		0.86 [0.22, 3.27]		0.95 [0.24, 3.79]
Kono		1.00		1.00
Limba		1.14 [0.28, 4.73]		1.23 [0.28, 5.41]
Mende		0.94 [0.32, 2.73]		0.96 [0.32, 2.84]
Temne		0.61 [0.19, 1.99]		0.67 [0.19, 2.43]
Korankoh		1.28 [0.22, 7.39]		1.56 [0.27, 9.00]
Other		0.86 [0.22, 3.27]		0.97 [0.26, 3.61]
**Partner’s educational attainment**				
No education		1.00		1.00
Primary		0.83 [0.42, 1.62]		0.83 [0.43, 1.61]
Secondary/higher		0.54^*^ [0.33, 0.88]		0.54^*^ [0.33, 0.89]
**Ideal number of children**				
0–3		1.00		1.00
4–5		1.12 [0.61, 2.03]		1.14 [0.63, 2.08]
≥6		8.03^***^ [4.54, 14.20]		8.10^***^ [4.58, 14.35]
**Contraceptive use**				
No		1.00		1.00
Yes		2.14^**^ [1.33, 3.42]		2.09^**^ [1.28, 3.41]
**Sex of household head**				
Male			1.00	1.00
Female			0.86 [0.57, 1.30]	0.84 [0.55, 1.29]
**Wealth index**				
Poorest			1.00	1.00
Poorer			1.59^*^ [1.04, 2.41]	1.70^*^ [1.07, 2.72]
Middle			1.80^*^ [1.15, 2.81]	2.09^**^ [1.29, 3.41]
Richer			1.01 [0.53, 1.91]	1.71 [0.84, 3.46]
Richest			0.28^**^ [0.11, 0.68]	0.62 [0.23, 1.67]
**Place of residence**				
Urban			1.00	1.00
Rural			1.82 [0.97, 3.43]	1.82 [0.94, 3.52]
**Province**				
Eastern			1.00	1.00
Northern			0.45^**^ [0.27, 0.76]	0.39^*^ [0.17, 0.87]
Northwestern			0.96 [0.53, 1.75]	0.97 [0.39, 2.38]
Southern			0.69 [0.40, 1.19]	0.60 [0.32, 1.11]
Western			0.46 [0.20, 1.03]	0.69 [0.26, 1.84]
**Random effect model**				
PSU variance (95% CI)	5.60 [4.19, 7.47]	4.89 [3.66, 6.53]	4.21 [3.19, 5.54]	4.62 [3.47, 6.15]
ICC	0.63 [0.56, 0.69]	0.60 [0.53, 0.67]	0.56 [.49, 0.63]	0.58 [0.51, 0.65]
N	2253	2253	2253	2253
Number of clusters	561	561	561	561

aOR: adjusted OR; ICC: intraclass correlation coefficient; PSU: primary sampling unit.

*p<0.05.

**p<0.01.

***p<0.001.

1.00=reference category.

#### Random effect results

Table 3 indicates considerable variations in the factors associated with high completed fertility among married and cohabiting women in Sierra Leone among the clusters (σ2=5.60, 95% CI 4.19 to 7.47) in model I. Approximately 63% of the prevalence of high completed fertility was attributed to the variations between the clusters (ICC=0.63). The between-cluster difference decreased to 60% in model II, was reduced to 56% in model III and increased to 58% in model IV. These ICC results suggest that the likelihood of high completed fertility variations can be attributed to the variances across the clusters.

## Discussion

Our study examined the provincial distribution and predictors of high completed fertility among married and cohabiting women aged 40–49 y in Sierra Leone. A significant proportion (>60%) of married and cohabiting women aged 40–49 y in Sierra Leone had high completed fertility. The individual-level factors associated with high completed fertility include partner’s education, ideal number of children and use of contraceptives, whereas wealth index and province were the contextual factors associated with high completed fertility among married and cohabiting women in Sierra Leone.

The study revealed that a significant proportion (>60%) of married and cohabiting women aged 40–49 y in Sierra Leone has high completed fertility. One possible reason for high completed fertility among women in Sierra Leone could be lower levels of education, which may limit access to family planning information and resources.^29^ Cultural norms that favour large families can influence these desires. In some cultures, having a large family is associated with higher social status or prestige.^30^ Lack of awareness or misconceptions about family planning methods and their effectiveness can hinder their use.^31^ The current study suggests that a multifaceted approach that addresses social, economic and cultural factors is probably needed to significantly reduce high completed fertility.

Regarding province, the study found that married and cohabiting women in Sierra Leone's Northern province have lower odds of high completed fertility compared with the Eastern province. The Northern province might have experienced different economic development patterns than the Eastern province. Lower poverty levels or better access to education in the North could be contributing factors.^15^ The types of livelihood available in each province might influence fertility desires. For example, if agriculture dominates in the East and requires more child labour, couples might desire larger families. Cultural practices related to marriage, family structure or gender roles might vary between provinces.^30^ For example, if the North has a more robust tradition of extended families, couples might feel less pressure to have several biological children. The availability and accessibility of family planning services and information might be better in the Northern province compared with the East.^15^ This could lead to higher contraceptive use rates and lower fertility. Social attitudes towards family planning could differ between provinces. Greater acceptance and openness in the North could encourage the use of contraceptives.

The current study revealed that women in Sierra Leone with partners who have secondary or higher education are less likely to have high completed fertility compared with those whose partners have no formal education. Partners with higher education might have better access to information and knowledge about family planning methods and their benefits.^32^ They might be more likely to discuss these options with their partners and encourage their use. Higher education could be associated with better communication skills within couples. This could lead to more open and honest discussions about family size preferences, the desired number of children and family planning methods.^33,34^

In our study, women in Sierra Leone desiring ≥6 children are more likely to have high completed fertility compared with those wanting 0–3 children, which is quite intuitive. Still, there could be underlying reasons influencing this desire for larger families. Women who desire ≥6 children probably prefer larger families.^35^ This preference can be influenced by cultural norms, religious beliefs or a perceived need for an extensive support network.^36^ Women who desire large families might have misconceptions about family planning methods or their effectiveness.^37^ This could lead to inconsistent use or avoidance altogether.

Women in Sierra Leone who were using contraceptives at the time of the survey had a higher likelihood of high completed fertility. The use of contraceptives might not always be for complete fertility prevention but for birth spacing.^38^ Women might desire some time between births, but still want to achieve a large family size overall. The availability of different contraceptive methods can vary. If women have limited access to more effective long-acting reversible options like intrauterine device or implants, they might rely on less effective methods with higher failure rates.^38^

The current study revealed that women in Sierra Leone who are in the poorer and middle wealth index quintiles have higher odds of high completed fertility than those in the poorest wealth index quintile. Poorest women may have low fertility because of the lack of resources to take care of them.^39^ Again, such women the poorest women often face the highest levels of child mortality due to inadequate healthcare, nutrition, and living conditions.^40,41^ As a result, while they may give birth to many children, not all survive to adulthood. This reduces the number of surviving children and, thus, completed fertility may appear lower compared to slightly better-off women who experience fewer child deaths.

## Policy and practice implications

Based on these findings, the government and policymakers in Sierra Leone should expand access to family planning services and a wider variety of contraceptive methods across all provinces, particularly in areas with lower education levels and higher desired family sizes. Educational programmes on family planning methods, their effectiveness and healthy family planning practices should be implemented. These should target both men and women, potentially through schools, community health centres and media campaigns. Misconceptions surrounding the use of contraceptives must be addressed. This could involve promoting consistent and proper use and differentiating between birth spacing and prevention methods. The government and policymakers should invest in girls' education, which can lead to lower desired family size and empowering women to make informed choices about their reproductive health. Men should be engaged in family planning discussions and education initiatives. Their understanding and support can significantly impact fertility decisions. Provincial-specific family planning programmes that address each area's unique needs and cultural contexts (e.g. the Northern province might require different approaches compared with the Eastern province) should be developed. The government and policymakers should address poverty through economic development initiatives. Improved living standards might decrease the desired family size, especially if smaller families are seen as less of a burden. Programmes that enhance child health and survival rates must be invested in. Couples might feel less pressure to have several children when children are more likely to survive childhood. By implementing these policies and practices, Sierra Leone can address high completed fertility and empower women to make informed choices about their reproductive health.

## Strengths and limitations

One of the key strengths of the current study is the use of the 2019 SLDHS, a large-scale survey designed to represent the entire country. This allows for generalisable findings about fertility among married and cohabiting women aged 40–49 y in Sierra Leone. The SLDHS follows standardised methods for data collection, ensuring consistency and allowing for comparisons with other countries that have conducted similar surveys. The SLDHS collects data on various demographic and health indicators, including education, wealth index, family planning practices and desired family size. This allows for the exploration of multiple factors associated with high fertility. However, the current study has some limitations. The SLDHS is a cross-sectional survey, meaning that it captures data simultaneously. This limits the ability to establish causal relationships between identified factors and high completed fertility. Reliance on self-reported data can introduce recall and social desirability bias. Additionally, because the dataset used is secondary from a survey, we were not able to account for certain important factors, such as cultural and religious beliefs, access to reproductive healthcare services, employment type, gender roles and empowerment, among others.

## Conclusions

A significant proportion (>60%) of married and cohabiting women aged 40–49 y in Sierra Leone has high completed fertility. Partner’s education, the ideal number of children, the use of contraceptives and wealth index, as well as province, were the individual and contextual factors respectively associated with high fertility among women in Sierra Leone. These findings underscore the complexity of fertility decisions and highlight the importance of context-specific interventions. Building on our findings, policymakers in Sierra Leone must increase access to and education on family planning methods to empower women to make informed choices about their fertility. Educational opportunities, particularly for men, must be supported, because they are usually the household heads, and can influence fertility decisions. The government should implement policies and programmes that encourage male involvement in family planning, given that the decision-making process regarding the use of contraceptives among women in Sierra Leone is often a joint one with their husbands. To optimize family planning programmes, tailored family planning programmes should be developed to address the specific needs of different provinces within Sierra Leone. Addressing cultural norms around ideal family size through educational campaigns and community engagement is essential. Future research could benefit from conducting in-depth interviews with women who use contraception to understand their motivations and experiences. Additionally, addressing the underlying factors associated with wealth and fertility, such as poverty reduction and promoting child well-being, can contribute to reducing desired family size in Sierra Leone. Moreover, understanding power dynamics within couples and households regarding contraceptive decision-making would provide valuable insights. Understanding these dynamics is crucial for developing more targeted and effective interventions that empower women to make informed reproductive health decisions.

## Data Availability

The data used for this study are freely available at http://dhsprogram.com/data/available-datasets.cfm.
